# A Gluten-Free Diet during Pregnancy and Early Life Increases Short Chain Fatty Acid-Producing Bacteria and Regulatory T Cells in Prediabetic NOD Mice

**DOI:** 10.3390/cells12121567

**Published:** 2023-06-06

**Authors:** Valdemar Brimnes Ingemann Johansen, Daisy Færø, Karsten Buschard, Karsten Kristiansen, Flemming Pociot, Pia Kiilerich, Knud Josefsen, Martin Haupt-Jorgensen, Julie Christine Antvorskov

**Affiliations:** 1 Department of Pathology, Bartholin Institute, Rigshospitalet, 2100 Copenhagen, Denmark; daisyfaro@gmail.com (D.F.); buschard@dadlnet.dk (K.B.); knud@eln.dk (K.J.); martin.haupt-joergensen@regionh.dk (M.H.-J.); 2 Laboratory of Genomics and Molecular Biomedicine, Department of Biology, August Krogh Building, University of Copenhagen, Universitetsparken 13, 2200 Copenhagen, Denmark; kk@bio.ku.dk (K.K.); pkii@ssi.dk (P.K.); 3 Steno Diabetes Center, Borgmester Ib Juuls Vej 83, 2730 Herlev, Denmark; flemming.pociot@regionh.dk; 4 Department for Congenital Disorders, Danish Center for Neonatal Screening, Statens Serum Institut, 2300 Copenhagen, Denmark

**Keywords:** type 1 diabetes, gut microbiota, regulatory T cells, short chain fatty acids, autoimmunity, dietary factors

## Abstract

The incidence of the autoimmune disease type 1 diabetes is increasing, likely caused by environmental factors. A gluten-free diet has previously been shown to ameliorate autoimmune diabetes in non-obese diabetic (NOD) mice and humans. Although the exact mechanisms are not understood, interventions influencing the intestinal microbiota early in life affect the risk of type 1 diabetes. Here, we characterize how NOD mice that are fed a gluten-free (GF) diet differ from NOD mice that are fed a gluten-containing standard (STD) diet in terms of their microbiota composition by 16S rRNA gene amplicon sequencing and pancreatic immune environment by real-time quantitative PCR at the prediabetic stage at 6 and 13 weeks of age. Gut microbiota analysis revealed highly distinct microbiota compositions in both the cecum and the colon of GF-fed mice compared with STD-fed mice. The microbiotas of the GF-fed mice were characterized by an increased *Firmicutes*/*Bacteroidetes* ratio, an increased abundance of short chain fatty acid (particularly butyrate)-producing bacteria, and a reduced abundance of Lactobacilli compared with STD mice. We found that the insulitis score in the GF mice was significantly reduced compared with the STD mice and that the markers for regulatory T cells and T helper 2 cells were upregulated in the pancreas of the GF mice. In conclusion, a GF diet during pre- and early post-natal life induces shifts in the cecal and colonic microbiota compatible with a less inflammatory environment, providing a likely mechanism for the protective effect of a GF diet in humans.

## 1. Introduction

Type 1 diabetes (T1D) is an autoimmune T cell-driven disease, with increasing incidence in the Western world [1] that cannot be explained by genetic factors alone but rather by changes in the exposure to environmental factors affecting the development of the disease. Studies have demonstrated that gluten can impact the onset of autoimmune diabetes in mouse models. For example, when non-obese diabetic (NOD) mice were switched to a gluten-free (GF) diet after weaning, the incidence of autoimmune diabetes decreased from 64% to 15% [2]. Increasing evidence shows that there is a change in the microbiota and the immune reactivity of patients with T1D before disease onset [3,4]. Some bacteria, viruses, and parasites are thought to educate the immune system and thereby prevent autoimmunity [5], and the microbiome is essential in maintaining the intestinal homeostasis and mucosal barrier [6]. Increased gut permeability causes intestinal immune cell activation, as demonstrated in mice where the loss of gut barrier continuity leads to the activation of islet-specific T cells within the intestinal mucosa and to autoimmune diabetes [7].

Importantly, pancreatic *β*-cell depletion, as a consequence of the loss of immunological tolerance, has been described to be regulated by gluten-induced enteropathy [8] and innate immunity [9]. In spontaneous diabetic bio-breeding (BB) rats, the proportion of Th1 cells within the mesenteric lymph nodes proliferate specifically in response to wheat protein antigens [10]; a GF diet could reverse this response in the gut, characterized by an anti-inflammatory state with more transforming growth factor-*β* (TGF-*β*)-producing T cells [11]. Interestingly, in gluten intervention studies, the cytokine profiles of mucosa-associated lymphoid tissue in the gut with high IFN*γ* production are associated with the incidence of diabetes in animals [8,12,13,14].

A GF diet also has an important impact on the development of T1D in humans. For example, a GF diet introduced to newly diagnosed T1D patients is associated with a significantly better disease outcome assessed by HbA1c and insulin dose-adjusted HbA1c (IDAA1c) [15]. Likewise, a high gluten intake by mothers during pregnancy could increase the risk of their children developing T1D [16].

The aim of this study was to characterize the effect of a GF diet during pre- and postnatal life on the microbiota composition in the colon and cecum of NOD mice and to investigate whether specific changes correlated with T cell infiltration of the pancreas compared with STD-fed mice.

## 2. Materials and Methods

### 2.1. Ethics

Experiments were approved by the Animal Experiments Inspectorate (2012-15-2934-00086) and regulated by Directive 2010/63/EU on the protection of animals used for scientific purposes.

### 2.2. Animals and Diets

**Figure 1:**
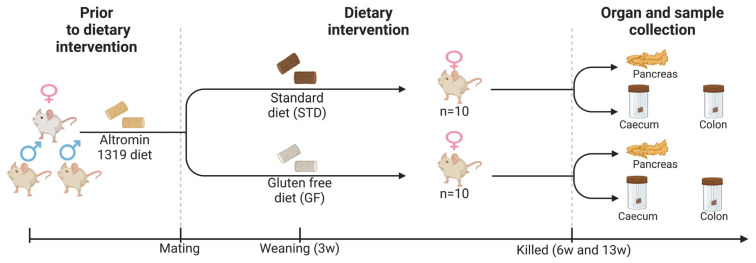
**Schematic of the dietary intervention study**. Female NOD mice were divided into two groups and were fed either a gluten-containing standard diet or a gluten-free diet at the time of mating and throughout life. Their offspring (*n* = 10 in each group) followed the maternal dietary intervention. Mice were sacrificed at 6 or 13 weeks of age, when gut stool from both cecum and colon were collected (as well as pancreas).

NOD/BomTac mice (Taconic, Germantown, NY, USA) were acclimatized on an Altromin 1319 diet (Altromin, Lage, Germany) until the initiation of breeding (2 males and 1 female per cage) from 7 weeks of age. From the time of mating and throughout the female offspring’s weaning period and life, the mice were fed ad libitum either a GF-modified Altromin diet [17] or a gluten-containing standard (STD) Altromin diet [17] (Figure 1). The mice were kept in a specific pathogen-free (SPF) environment in cages with 2–8 mice with a 12 h light cycle, humidity of 55 ± 10% with 16 air changes per hour, and temperature of 22 °C (±2 °C). Shelter and nesting materials were provided in all cages. The 6- and 13-week-old female offspring were weighted, their blood glucose levels were measured from tail blood with a FreeStyle Lite Glucometer (Abbott, Abbott Park, IL, USA), and they were sacrificed by cervical dislocation followed by extirpation of feces, blood, and organs (Figure 1).

### 2.3. Islet Autoantibodies

The quantification of serum islet autoantibodies (IAAs) titers was performed using a mouse insulin auto-antibodies ELISA kit (Abbexa, Cambridge, UK) according to the manufacturer’s protocol. Plates were read on a Tecan Sunrise plate absorbance reader (Tecan, Grödig, Austria) at 450 nm. Samples were diluted 1:100 prior to quantification.

### 2.4. Histology

Half of each pancreas was fixed in 10% formalin, pH 7.4, and embedded in paraffin. Blocks were cut in 4 μm sections at three depths and stained with hematoxylin and eosin (HE). Slides were cut 150 μm apart to avoid scoring the same islet more than once. Slides were randomized and blinded. The degree of lymphocyte infiltration was evaluated using a light microscope (Olympus, Ballerup, Denmark) and graded as follows: 0, no infiltration; 1, intact islets but with few mononuclear cells surrounding the islets; 2, peri-insulitis; 3, islet infiltration <50%; 4, islet infiltration >50%.

### 2.5. Quantitative PCR

Half a pancreas was snap frozen in liquid nitrogen and stored at −80 °C. Tissue was homogenized for 10 s at medium speed with the Polytron PT 10–35 tissue homogenizer (Kinematica, Malters, Switzerland). RNA isolation was performed using the TRIzol (Invitrogen, Carlsbad, CA, USA) procedure. RNA concentration and purity was measured using a NanoDrop 1000 spectrophotometer (Thermo Scientific, Waltham, MA, USA). Samples were diluted to a concentration of 300–500 ng/μL and stored at −20 °C. cDNA synthesis was performed using the qScript cDNA Supermix (Quanta Biosciences, Beverly Hills, CA, USA). RT-qPCR was carried out on a Ligthcycler II (Roche, Basel, Switzerland) in Lightcycler capillaries (Roche, Basel, Switzerland). RT-qPCR reactions mixes contained 4 μL cDNA, 1 μL primer mix 10 μM, 2 μL 5× master mix (FastStart Taq DNA polymerase, reaction buffer, MgCl_2_, SYRB green I dye and dNTP mix), and 3 μL sterile nuclease-free water. Serial dilutions with sterile water of the target standard fragment were used as the standard. A melting curve was performed to verify specificity of the product. CyPa was used to normalize the RT-qPCR results of the selected targets.

### 2.6. Gut Microbiota

Total DNA was extracted from both cecum and colon feces samples of the mice and subjected to 16S rRNA gene amplicon sequencing, targeting the hypervariable V4 region. Sequence data were analyzed using R. Using the DADA2 pipeline, forward and reverse sequences were matched and ordered. Quality plots showing error rates by sequence lengths were assessed and reads were trimmed at 240 and 140 base pairs for forward and reverse sequences, respectively, ensuring an adequate overlap. Reads were dereplicated and additional quality checks applied to denoise samples and increase accuracy. Sequences that diverged from the target 230–234 base pairs in length were removed. Evaluation of the chimeric sequences showed 86.3% to be non-chimeric. The remaining 13.7% chimeric sequences were taken out and further analyses were conducted on the non-chimeric sequences. To assign taxonomy, a neighbor-joining phylogenetic tree was constructed via the ClustalW method from non-chimeric sequences, using the Silva reference database version 128. In total 981 amplicon sequence variants (ASVs) were identified and annotated to the highest possible taxonomy level. Samples were ascribed metadata comprising mouse number, age, diet, and sample site. Beta diversity was subsequently investigated, as well as correlation analysis and comparison of individual bacterial reads. To find the closest species of specific ASVs, the Molecular Evolutionary Genetics Analysis Version 7.0 (MEGA7) for bigger dataset software was used to align sequences and to construct phylogenetic trees. The hierarchy browser in the Ribosomal Database Project (RDP) database (Michigan State University) was applied to find rRNA sequences from related species. The related sequences from the RDP database were selected by taking all species belonging to the level, which was endorsed by the DADA2 pipeline and Silva database, or one level above this endorsed level. Sequences were thereafter aligned using MUSCEL to find the closest species match and the bootstrap method with 500 replications was applied to evaluate the strength of the match.

### 2.7. Statistical Analysis

GraphPad Prism version 9.01 (GraphPad Software, San Diego, CA, USA) was used for statistical analysis and *p* values < 0.05 were considered statistically significant. Parametric two-tailed unpaired *t*-tests were performed to test the significance of the differences between the diet groups and the age groups in insulitis mean scores, relative expression level of genes in qPCR, body weight, blood glucose, IAAs, Firmicutes/Bacteroidetes ratios, and abundance of SCFA-producing bacteria. Abundance of bacteria from the *Lactobacillus* species was analyzed by nonparametric Mann-Whitney U test. First, data were checked for normality by D’Agostino and Pearson normality tests and qq-plots. In cases where data were not normally distributed, Mann–Whitney tests were performed. When differential insulitis scores were analyzed as independent scores, two-way ANOVA was performed in SPSS (SPSS, software, SPPS, Inc., Chicago, IL, USA). For analysis of differential abundance (Table 1), *p*-values were calculated by two-tailed unpaired *t*-test and q-values as calculated by the FDR method of Benjamini, Krieger, and Yekutieli. Bacterial groupings with less than 70% representation of the NOD mice in either of the diet groups were excluded. Significance was designated with the following for two-tailed unpaired Student’s *t*-tests: * *p* ≤ 0.05, ** *p* ≤ 0.01, *** *p* ≤ 0.001, and **** *p* ≤ 0.0001. Data are reported as means ±SEM.

## 3. Results

### 3.1. A Gluten-Free Diet Reduces Insulitis Degrees and Increases the Expression of FOXP3 and GATA3

In this study, we aimed at exploring the effects of a pre- and postnatal GF diet on T1D development in vivo by analyzing NOD mice undergoing dietary intervention until 6 and 13 weeks of age. We first sought to determine whether a GF diet in NOD mouse offspring, of mothers fed a GF diet from the time of mating, influenced the degree of insulitis and immune markers of the pancreas (Figure 1).

**Figure 2:**
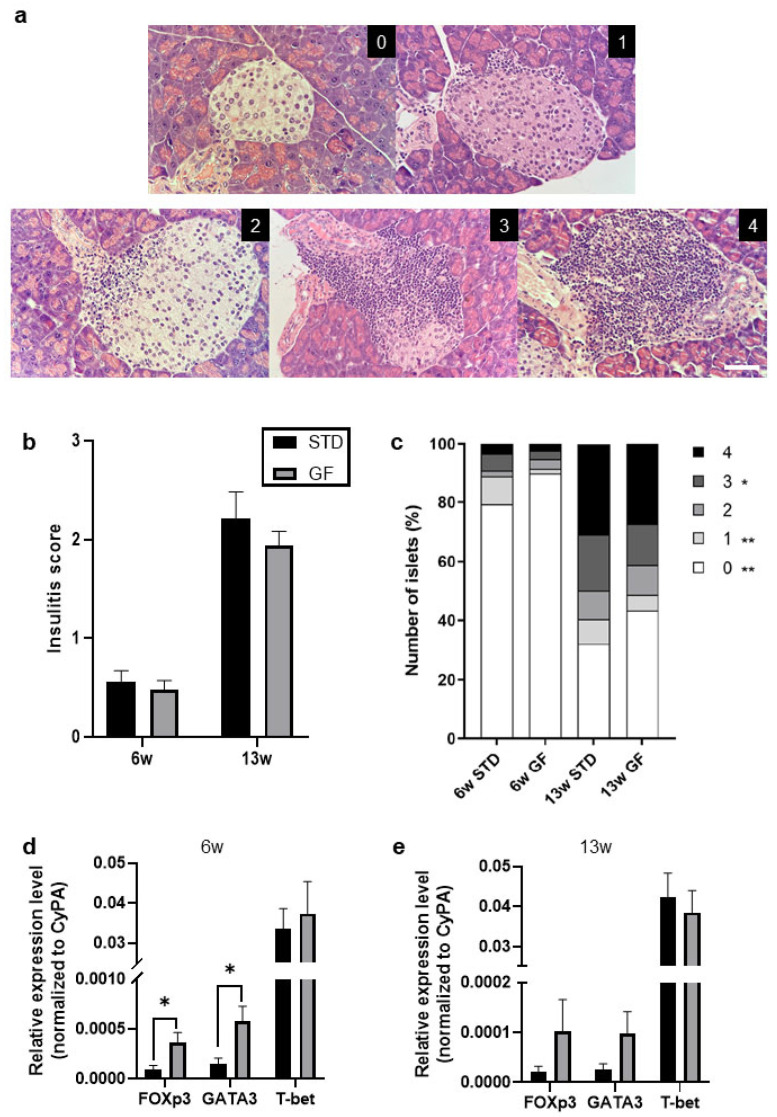
**A gluten-free diet during pre- and postnatal life affects the expression of regulatory T cell and T helper 2 cell markers**. (**a**) Representative hematoxylin/eosin images of different degrees of insulitis within the pancreatic islets of Langerhans. Islet infiltration was graded as follows: 0, no infiltration; 1, intact islets but with few mononuclear cells surrounding the islets; 2, peri-insulitis; 3, islet infiltration <50%; 4, islet infiltration >50%. This scoring system was utilized as a reference throughout the analysis in (**b**,**c**). Scale bar indicates 100 µm. (**b**,**c**) The degree of insulitis was scored in the pancreas from mice at 6 and 13 weeks of age. Data shown are means (**b**) or the differential grading (**c**) of the insulitis scores ±SEM. Statistics in (**b**) are Student’s *t*-tests. Statistics in (**c**) are 2 way ANOVA. (**d**,**e**) Relative expression of FOXp3, GATA3, and T-bet of mice from a at either 6 (**d**) or 13 (**e**) weeks of age. * *p* ≤ 0.05, ** *p* ≤ 0.01.

While the mean insulitis scores (for representative images of grading scale, see Figure 2a) of NOD mice fed a GF diet did not differ significantly compared with NOD mice fed the STD diet until 6 weeks and 13 weeks of age (Figure 2b), we observed significantly lower immune cell infiltration in the pancreatic islets of Langerhans in GF mice compared with STD mice, when scores 0, 1, and 3 were considered (Figure 2c). 

**Figure 3:**
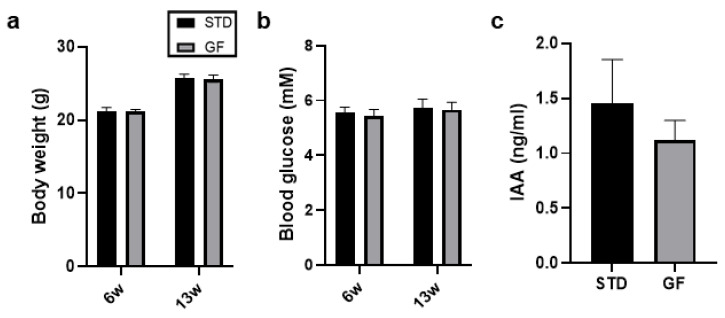
**NOD mice undergoing dietary intervention are prediabetic.** (**a**–**c**) Body weight (**a**), blood glucose (**b**), or islet auto-antibodies (**c**) of mice from the two dietary arms in Figure 1 were noted and reported. Statistics in (**a**–**c**) are Student’s *t*-tests, and data shown are means ±SEM.

Although we found a similar trend for FOXp3 and GATA3 at 13 weeks of age, the differences were not significant between the GF- and STD-fed NOD mice (Figure 2e), suggesting that the immune modulating effect of a GF diet during pre- and postnatal life decreases with age in these mice. Of note, the NOD mice undergoing the different dietary restrictions in this study did not differ significantly in body weight (Figure 3a), blood glucose levels (Figure 3b), or islet auto-antibodies (IAAs) (Figure 3c); they were thus prediabetic.

Interestingly, the expression of FOXp3 in the pancreas, a marker of regulatory T cells shown to suppress autoimmune diabetes [18], was increased in the GF mice compared with the STD mice (Figure 2d). T-bet, known to control genetic programs that coordinate type 1 immune responses [19], did not differ in isolated pancreatic tissue when GF- and STD-fed mice were compared (Figure 2d). However, the master regulator of Th2 differentiation, GATA3, was significantly upregulated in the pancreas of the GF-fed mice (Figure 2d), indicating that the Th1/Th2 balance was decreased in the GF-diet-fed NOD mice compared with the STD-diet-fed mice, an imbalance that might contribute to a distinct autoimmune response in these mice [20].

### 3.2. Altered Beta Diversity of Gut Microbiota from NOD Mice Fed a Gluten-Free Diet

**Figure 4:**
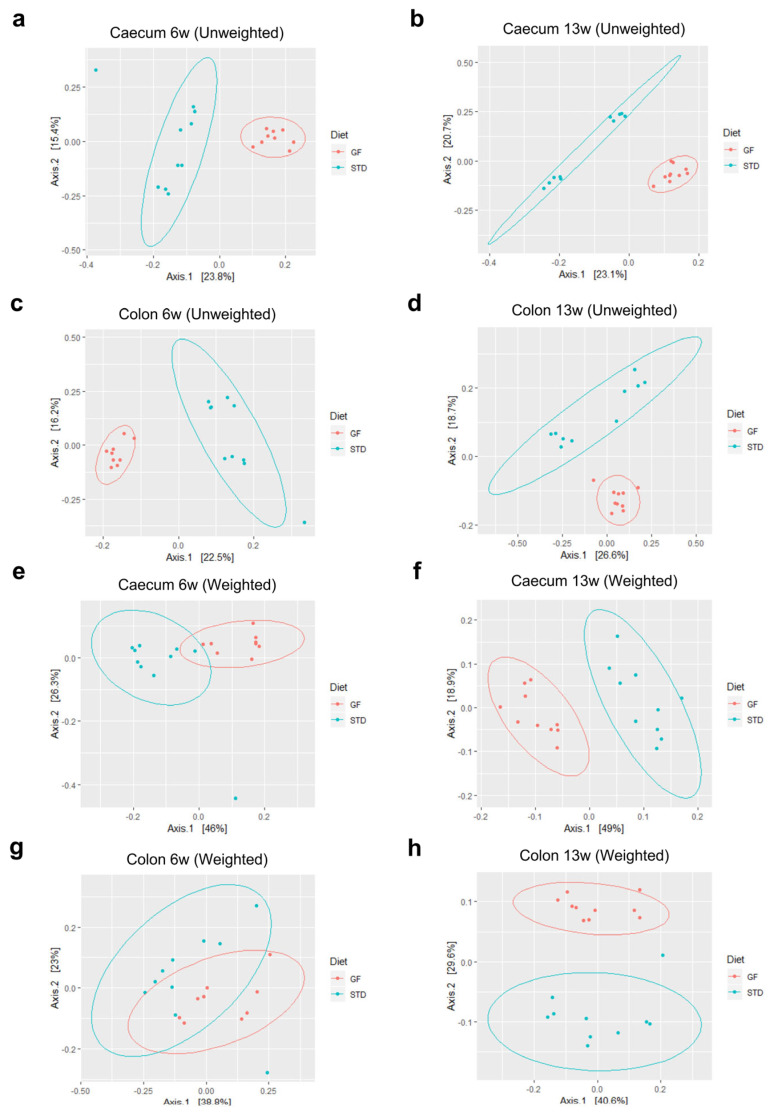
**Mice fed a gluten-free diet present with diet-induced alterations in the intestinal microbiota compositions: PCoA analysis of cecal and colonic stool samples**. (**a**–**h**) Cecal (**a**,**b**,**e**,**f**) or colonic (**c**,**d**,**g**,**h**) stool samples from 6-week- (**a**,**c**,**e**,**g**) or 13-week- (**b**,**d**,**f**,**h**) old NOD mice fed either a gluten-free (red) or standard (green) diet were analyzed for beta diversity by unweighted (**a**–**d**) or weighted (**e**–**h**) unique fraction metrics (UniFrac) and permutational multivariate analysis of variance (PERMANOVA).

Studies have demonstrated that a diet low in gluten can elicit alterations in the composition of the human intestinal microbiome [21,22]; changes in gut microbiota have been shown to influence the development of autoimmune diabetes [23,24]. This prompted us to investigate potential differences in the colonic and cecal microbiota of NOD mice fed either a GF or an STD diet. We first asked whether the unweighted unique fraction metric (UniFrac), which takes low-abundance species into account, differed between the two groups by permutational multivariate analysis of variance (PERMANOVA). Importantly, 16s rRNA gene amplicon sequencing and subsequent DADA2-pipeline analysis of samples from the cecum (Figure 4a,b) (6 and 13 weeks R2 = 0.22) and colon (Figure 4c,d) (6 weeks R2 = 0.21, 13 weeks R2 = 0.19) of NOD mice exhibited significantly distinct (*p* < 0.001) distribution patterns, as visualized by principal component analysis (PCoA) plots. 

By performing PERMANOVA from weighted UniFracs, we then took high-abundance species into account to describe changes in stable Amplicon sequence variants (ASVs). Interestingly, here we also observed uniquely allocated distributions of both cecal (Figure 4e,f) and colonic (Figure 4g,h) microbiomes in a manner dependent on the diet. The proportion of variation in the microbial composition (diet as the grouping variable) within the cecum was R2 = 0.36 at 6 weeks of age (*p* < 0.001) and R2 = 0.44 at 13 weeks of age (*p* < 0.001). Similarly, colonic samples had R2 = 0.20 at 6 weeks of age (*p* < 0.004) and R2 = 0.27 at 13 weeks of age (*p* < 0.001). These data corroborate that a GF diet during pre- and postnatal life changes the beta diversity of low- and high-abundance species of NOD mice early in life at distinct sites in the gastrointestinal tract.

### 3.3. A GF Diet Changes the Gut Microbiome in NOD Mice

Of 69 identified genera, the abundances of four and eight bacterial families differed significantly in the cecum of the NOD mice in a manner dependent on the diet at both 6 and 13 weeks of age (Table 1). Similarly, the abundances of six and five bacterial families were significantly different in the colon of these mice depending on the diet at 6 and 13 weeks of age, respectively (Table 1). Among these families, *Clostridales* bacteria, which are associated with the induction of regulatory T cells [25] and are considered anti-inflammatory [26,27,28], were significantly upregulated in the colon and cecum of GF mice compared with STD mice at both 6 and 13 weeks of age (Table 1). 

**Figure 5:**
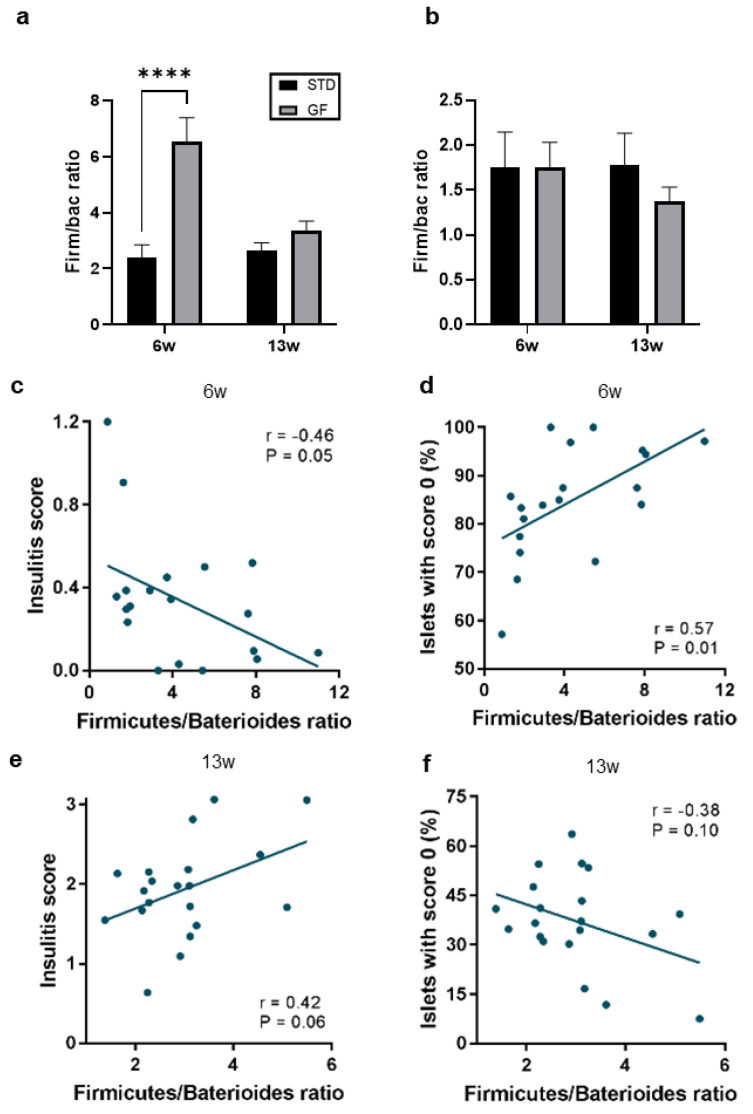
**Firmicutes/Bacteroidetes ratios are increased in the cecum of mice fed a gluten-free diet and correlate with decreased insulitis scores**. (**a**,**b**) Firmicutes/Bacteroidetes ratios of cecal (**a**) or colonic (**b**) stool samples from mice at 6 or 13 weeks of age fed either a standard or gluten-free diet. Data shown in (**a**,**b**) are means ±SEM. Statistics are Student’s *t*-test. **** *p* ≤ 0.0001. (**c**–**f**) Spearman’s correlation tests of insulitis scores (**c**,**e**) and islets with no insulitis (**d**,**f**) and Firmicutes/Bacteroidetes ratios in the cecum mice in the dietary intervention study at 6 weeks (**c**,**d**) or 13 weeks (**e**,**f**) of age. Spearman’s coefficient (r) and complementary *p* values are indicated in each panel.

On the contrary, *Bacteriodales* bacteria, which correlate positively with a high frequency of diabetogenic iNKT17 cells [29], were significantly upregulated in the cecum of STD mice compared with GF mice at both 6 and 13 weeks of age (Table 1). Changes in the microbial composition may play a significant role in autoimmunity [30], as these can contribute to the loss of immune tolerance [31,32]. More specifically, a decreased Firmicutes/Bacteroidetes ratio has been suggested as a marker of immune-mediated inflammatory diseases [33] and T1D [34]. At 6 weeks of age, we found that the Firmicutes/Bacteroidetes ratio was significantly lower in the cecum of STD mice compared with GF mice (Figure 5a), an effect that was dependent on the sampling site of the gastrointestinal tract (Figure 5b). It is well known that the microbiome changes with age [35], which was also evident in data from the present study (Figure 5a,b). Interestingly, the insulitis scores of mice in this study (Figure 5b,c) tended to associate negatively with the cecal Firmicutes/Bacteroidetes ratios (Figure 5c) and the proportion of mice with islets of no insulitis correlated significantly with increased cecal Firmicutes/Bacteroidetes ratios (Figure 5d). This correlation was not seen in the colon of these mice (Figure 5e,f). In summary, the data indicated that microbial composition changes in the gastrointestinal tract of NOD mice depended on diet and age, affecting the Firmicutes/Bacteroidetes ratio and potentially protecting against autoimmunity.

### 3.4. Differential Abundance of Butyrate-Producing and Lactobacillus Bacteria in GF and STD Mice Correlates with Insulitis

Many bacteria belonging to the *Clostridiales* order, such as *Lachnospiraceae* and *Ruminococcaceae*, produce butyrate [36], which has been shown to play a role in reducing glycemia [37], restoring homeostatic levels of inflammatory markers, reducing ROS production [38], and increasing the differentiation of β-cells and their production of insulin [39]. We were therefore interested in further characterizing possible differences between GF and STD NOD mice in terms of their intestinal abundances of butyrate-producing bacteria. 

**Figure 6:**
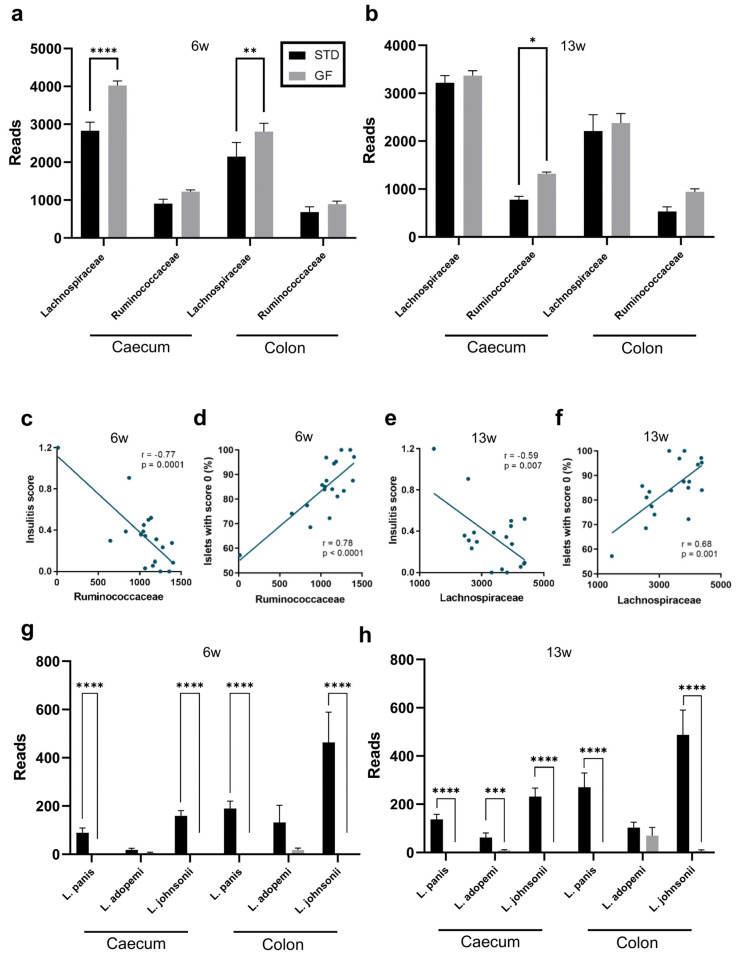
**Differential enrichments of butyrate-producing and *Lactobacillus* bacteria in mouse cecal and colonic microbiota are dependent on gluten and correlate with low insulitis score**. (**a**,**b**). Reads of butyrate-producing bacteria in cecal and colonic stool samples from 6- (**a**) or 13- (**b**) week-old NOD mice fed a standard or a gluten-free diet. Data shown are means ±SEM. Statistics are Student’s *t*-test. (**c**–**f**). Correlations of insulitis scores (**c**,**e**) and islets with no insulitis (**d**,**f**) and the reads of butyrate-producing bacteria in the cecum of mice in the dietary intervention study at 6 weeks of age. Plots are presented with Spearman’s coefficient (r) and complementary *p* values. (**g**,**h**). Presence of the most abundant *Lactobacillus* species in the cecum or colon of 6- (**g**) or 13- (**h**) week-old NOD mice fed a standard or gluten-free diet. Data shown are means ±SEM. Statistics are from a Mann–Whitney U test. * *p* ≤ 0.05, ** *p* ≤ 0.01, *** *p* ≤ 0.001, and **** *p* ≤ 0.0001.

At 6 weeks of age, we observed a significantly higher abundance of butyrate-producing bacteria in both the cecum and the colon of mice which had been GF throughout their life compared with STD mice (Figure 6a). Specifically, bacteria from the *Lachnospiraceae* family were significantly more abundant in the cecum and colon of GF mice; the abundance of bacteria belonging to *Ruminococcaceae* followed a similar trend (Figure 6a). Whereas a similar trend was observed at 13 weeks of age, only the population of *Ruminococcaceae* bacteria in the cecum came out significantly different when comparing sample means of abundances from GF and STD mice (Figure 6b). Importantly, the insulitis score (Figure 6c) and numbers of islets with no insulitis (Figure 6d) of animals in this study correlated with high significance negatively and positively with their abundance of *Ruminococcaceae* in the cecum of mice at 6 weeks of age, respectively. This observation also held true when analyzing the Pearson correlations with respect to their abundance of *Lachnospiraceae* (Figure 6e,f). In addition to bacterial families, such as *Bacteroidales S24-7* and *Family XIII,* which were significantly different between STD and GF mice in terms of abundance (Table 1), specific bacterial strains belonging to the *Lactobacillaceae* family were significantly more abundant in the gut of the STD mice compared with the GF mice (Figure 6g,h).

In summary, the observed differences in the abundance of some of the analyzed bacteria in the GI tract of GF and STD mice, such as butyrate-producing bacteria, appear related to insulitis and may potentially play a role in the development of autoimmune diabetes in these mice later in life. The findings further emphasize the potential importance of the gut microbiota in the pathogenesis of autoimmune diseases and implicate gluten as an important modulator.

## 4. Discussion

In this study, we found that a GF diet, introduced prenatally, changed the gut microbiomes and further had an impact on the lymphocyte infiltration in the pancreas and on the expression of transcription factors in T cells and thus provided a possible mechanism for the effect of a GF diet [16].

We observed that GF mice had significantly less T cell infiltration in their islets when analyzing the effect of diet on the insulitis scores. This indicates that a pre- and postnatal GF diet might protect against the development of autoimmune diabetes in NOD mice. We did not observe any statistical difference in glycemic levels or IAAs between STD and GF mice. This might be expected, considering that hyperglycemia begins around 15 weeks of age in female NOD mice [40,41]. We also found evidence to suggest an increased presence of regulatory T cells in the pancreas of GF mice compared with STD mice, an observation that has also been found in T1D patients [42] and mouse models of autoimmune diabetes [43]. This might imply that a pre- and postnatal GF diet is capable of facilitating a more tolerogenic environment in the pancreas, which is consistent with previous work [11,44]. However, some findings in mice [45] and in humans [46,47] do not support the hypothesis that gluten can accelerate the development of autoimmune diabetes. 

Earlier studies have shown that a time window may exist for when the effect of a GF diet is optimal. In NOD mice, the autoimmune diabetes incidence was reduced from 62.5% to 8.3% in mouse offspring [17] if their mothers were on a GF diet during pregnancy, even though offspring were on a gluten-containing diet. These studies were confirmed in humans, where the T1D incidence was reduced to half when the mothers had a low gluten intake during pregnancy [16]. This is supported by studies showing that the diabetogenic potential of gluten seems to be dependent on the time of introduction both in BB rats [48] and NOD mice [49]. This is consistent with human studies where the early introduction of gluten-containing cereals to children increases the risk of developing islet auto-antibodies [50].

In this study, we found that GF and STD mice had distinct gut microbiomes. More specifically, we found that more than 50% of the genera, which were significantly more abundant in the gut of GF mice, belonged to butyrate-producing bacteria. These bacteria have been implicated in decreasing gut permeability [51], inducing regulatory T cells [27], being more abundant in diabetes-protected NOD mice [52], and also protecting against diabetes in humans [53,54,55]. The finding that the *Lactobacillus* species were consistently less abundant in the gut of GF mice compared with STD mice adds to a heterogenous amount of the literature, where some data suggest a high abundance of *Lactobacillus* bacteria to be linked to an increased risk of T1D [52,55], whereas other data indicate the opposite [56,57,58]. Thus, more studies are needed to help decipher whether these bacteria are protecting against or facilitating the development of T1D. 

The Firmicutes/Bacteroidetes ratio was significantly lower in the cecum of STD mice compared with GF mice already at 6 weeks of age; a lower ratio at this site of the GI tract correlated with a higher insulitis score. These findings are consistent with mouse models of autoimmune diabetes, which have been associated with higher fecal Bacteroidetes counts and lower Firmicutes counts [52,59,60]. Interestingly, our observations also corroborate other areas of the literature, where 6-week-old mouse models of systemic lupus erythematosus (a systemic autoimmune disease) show a lower Firmicutes/Bacteroidetes ratio [61,62]. Importantly, the authors found that the particular change was associated with markedly elevated autoimmune markers, intestinal barrier dysfunction, and microbiome dysbiosis [61], changes that are described to be implicated in the development of T1D and islet autoimmunity [63]. However, some of the literature also points towards microbiota-independent dietary protection in GF-diet-fed mice. For example, recent findings suggest that a maternal GF diet, until the weaning of their pups, delayed autoimmune diabetes in both the dams and the offspring of untreated NOD mice and in mice treated with an antibiotic cocktail, eliminating most of the bacteria [64].

The finding from this and previous studies implicate that specific Firmicutes/Bacteroidetes ratios might directly or indirectly modulate the immune system of the host to regulate autoimmune responses and that a GF diet might partially affect this microbial dysbiosis. In addition, the Bacteroidetes phylum is the main group of Gram-negative bacteria in the gut microbiota [65] and these bacteria are producing the proinflammatory molecule lipopolysaccharide (LPS) [66]. Therefore, the correlation between a low Firmicutes/Bacteroidetes ratio and higher insulitis scores reported here might be explained by increased LPS in STD mice compared with GF mice, driving inflammation. LPS from bacteria belonging to the Bacteroidetes phylum has been described to silence TLR4 signaling [67,68] and induce proinflammatory Th17 cell differentiation [69,70], leading to autoimmune diabetes [71]. Yet, some recent evidence suggest that the Firmicutes/Bacteroidetes ratio might be increased in individuals with Graves’ disease and Graves’ orbitopathy, compared with healthy controls [72], indicating that these bacterial phyla might affect autoimmunity in a disease-specific fashion. Another likely explanation for how specific ratios and their outcomes in terms of autoimmune regulation depend on the disease in question is that the determining factor might well be the distribution of specific Gram-negative bacteria within the phyla rather than the overall abundance of the phylum itself. 

## 5. Conclusions

In conclusion, our work provides evidence that a GF diet during pre- and postnatal life may protect against the development of autoimmune diabetes in mice associated with a modulation of the gut microbiome to be enriched in short chain fatty acid-producing bacteria. This correlates with decreased insulitis scores, and the pancreas of NOD mice fed a GF diet become more anti-inflammatory with increased Treg and decreased Th1/Th2 ratio markers. However, further research is needed to determine the role of specific bacterial strains in regulating autoimmune responses in autoimmune insulitis.

## Figures and Tables

**Table 1:** **Summary of statistical differences in taxa among 6-week- and 13-week-old NOD mice fed a GF or an STD diet.** Amplicon sequence variants (ASVs) were related to the taxonomy of phyla, genus, family, and order; the sample mean counts at the level of families, which were significantly different between the GF and STD diet fed NOD mice, were reported. *p*-values as calculated by two-tailed unpaired *t*-test and q-values as calculated by FDR method of Benjamini, Krieger, and Yekutieli.

Family	Mean Abundance		Statistical Significance	
	GF	STD	*p*-Value	q-Value
**Cecum 6 weeks**				
*Lactobacillaceae*	5.1	270.9	<0.0001	0.0001
*Family_XIII*	18.6	6.8	0.0002	0.0014
*Bacteroidales_S24-7_group*	301.7	1499.2	0.0002	0.0014
*Lachnospiraceae*	4024.7	2832.9	0.0003	0.0014
**Cecum 13 weeks**				
*Lactobacillaceae*	8.2	452.3	<0.0001	<0.0001
*Porphyromonadaceae*	330.0	93.6	<0.0001	<0.0001
*Ruminococcaceae*	1320.3	778.8	<0.0001	<0.0001
*Bacteroidales_S24-7_group*	851.6	1490.6	0.0002	0.0008
*Desulfovibrionaceae*	148.0	36.9	0.0003	0.0011
*Family_XIII*	16.6	6.4	0.0052	0.0156
*Deferribacteraceae*	44.2	11.6	0.0098	0.0249
*Christensenellaceae*	4.1	1.1	0.0251	0.0499
**Colon 6 weeks of age**				
*Christensenellaceae*	6.8	0.0	0.0007	0.0146
*Clostridiales_vadinBB60_group*	122.0	39.9	0.0013	0.0146
*Porphyromonadaceae*	649.7	218.2	0.0028	0.0204
*Lactobacillaceae*	18.0	838.3	0.0053	0.0247
*Family_XIII*	68.0	17.0	0.0056	0.0247
*Alcaligenaceae*	21.9	4.0	0.0102	0.0375
**Colon 13 weeks of age**				
*Porphyromonadaceae*	490.1	112.5	<0.0001	0.0007
*Lactobacillaceae*	86.7	931.0	0.0002	0.0017
*Ruminococcaceae*	942.6	533.0	0.0020	0.0132
*Desulfovibrionaceae*	99.5	24.9	0.0024	0.0132
*Family_XIII*	39.5	11.6	0.0073	0.0270

## Data Availability

Data are contained within the article. Raw sequencing data can be delivered upon reasonable request.
